# Cardiolipin is an Optimal Phospholipid for the Assembly, Stability, and Proper Functionality of the Dimeric Form of NhaA Na^+^/H^+^ Antiporter

**DOI:** 10.1038/s41598-019-54198-8

**Published:** 2019-11-27

**Authors:** Abraham Rimon, Ramakanta Mondal, Assaf Friedler, Etana Padan

**Affiliations:** 1 Department of Biological Chemistry, Alexander Silberman Institute of Life Sciences, the Hebrew University of Jerusalem, Edmond J. Safra Campus, Givat Ram, Jerusalem, 91904 Israel; 2 Institute of Chemistry, the Hebrew University of Jerusalem, Edmond J. Safra Campus, Givat Ram, Jerusalem, 91904 Israel

**Keywords:** Biochemistry, Biological techniques

## Abstract

Cardiolipin (CL) was shown to bound to the dimer interface of NhaA Na^+^/H^+^ antiporter. Here, we explore the cardiolipin-NhaA interaction both *in vitro* and *in vivo*. Using a novel and straightforward *in-vitro* assay in which n-dodecyl β-D maltoside (DDM) detergent is used to delipidate the dimer interface and to split the dimers into monomers; the monomers are subsequently exposed to cardiolipin or the other *E. coli* phospholipids. Most efficient reconstitution of dimers is observed by cardiolipin. This assay is likely to be applicable to future studies of protein–lipid interactions. *In-vivo* experiments further reveal that cardiolipin is necessary for NhaA survival. Although less efficient phosphatidyl-glycerol (PG) can also reconstitute NhaA monomers to dimers. We also identify a putative cardiolipin binding site. Our observations may contribute to drug design, as human NhaA homologues, which are involved in severe pathologies, might also require specific phospholipids.

## Introduction

Na^+^/H^+^ antiporters are found in the membranes of nearly all eukaryotic and prokaryotic cells and are essential for homeostasis of intracellular pH, sodium ion concentration, and volume^1^. Prokaryotic antiporters are evolutionarily conserved, such that insights regarding Na^+^/H^+^ antiporters in prokaryotes can contribute to the understanding of human homologues that are implicated in disease, including NHA2, which is involved in essential hypertension and diabetes, and NHE1, which is implicated in heart failure and has long been a drug target^2^.

**Figure 1:**
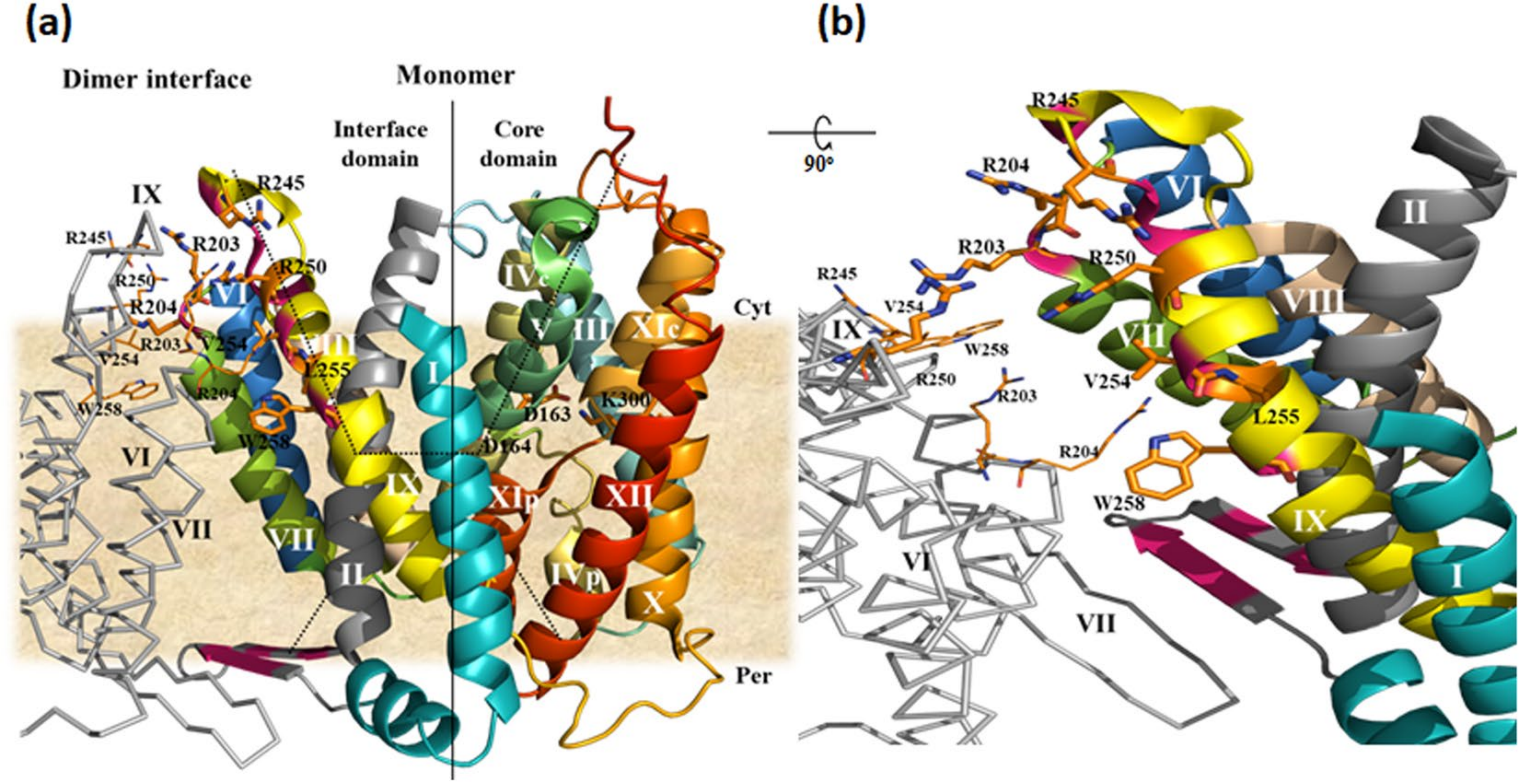
The NhaA dimer interface. (**a**) The crystal structure of the NhaA monomer and the interfacial domain between the two monomers of the NhaA dimer are represented according to^17^ (PDB.ID: 4ATV); one monomer is shown in colored ribbon, and the trans membrane segment (TM) are numbered by white Roman numerals. The relevant residues are in stick representation. Part of the other monomer of the dimer is shown in a grey line; the TMs are numbered in black, and the relevant residues are shown in line representation. In the NhaA dimer interface, sites where single Cys replacements on TM IX and the β-hairpin loop cross-link^22,26^ are marked in dark pink. The membrane is depicted in wheat color. The cytoplasmic funnel (dots) is composed of TMSs II, IVc (c and p denote cytoplasmic and periplasmic sides, respectively), V, and IX. The periplasmic funnel (dots) comprises TMs II, VIII and XIp. The TM IV/XI assembly of short helices connected by extended chains is shown together with the putative Na^+^ binding site (D163, D164). Cyt, cytoplasm; Per, periplasm. (**b**) The direct contacts between NhaA monomers^17^: W258 and V254 on TM IX of one monomer make a cross interface bridge to TM VII of the other monomer at R204. The two representations were generated using PyMOL with equal notifications.

NhaA, is the main Na^+^/H^+^ antiporter of *Escherichia coli*. It exchanges two protons for one sodium ion^3,4^, and it is very rapid and sensitive to pH, a property it shares with other prokaryotic and eukaryotic Na^+^/H^+^ antiporters^5^. The crystal structure of NhaA has provided numerous insights regarding the structure, mechanism of action and regulation by pH of Na^+^/H^+^ antiporters^6^. Specifically, the NhaA monomer comprises 12 transmembrane (TM) helices, packed in two domains: an interface domain, which connects two monomers of NhaA into a dimer (TMs I, II, VI–IX) (Fig. 1); and a core domain, which is involved in function (TMs III–V and X–XII). This structure represents a unique fold called the NhaA structural fold^7^. Despite very low homology between TMs III–V and TMS X–XII, they are topologically inverted repeats. Furthermore, TMs IV and XI are interrupted by extended chains that cross each other in the middle of the membrane (Fig. 1a). Because the extended chains are not completely hydrogen-bonded, they participate in the ion-binding site and create a very delicately balanced electrostatic environment in the middle of the membrane, which is crucial for the antiporter’s mechanism of action. At least six secondary transporters revealed to share the NhaA fold including non-homologues of NhaA^8–12^.

**Figure 2:**
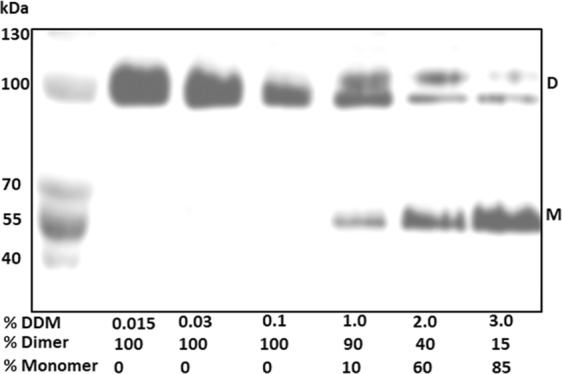
Increasing the DDM concentrations above 0.03% progressively splits native NhaA dimers into monomers. High-pressure membrane vesicles were isolated from TA16/pAXH3 cells expressing His-tagged NhaA, and the protein was affinity-purified and pre-incubated with increasing concentrations of DDM. Then, native gel sample-buffer was added, and the proteins were resolved on native gel. D, gel mobility of dimers. Notably, two bands were occasionally observed in the native gel, most likely due to different protein-Coomassie Blue complexes. M, gel mobility of monomers. The experiment was conducted three times with identical results.

Similarly to many membrane proteins, NhaA is a dimer in the native lipid membrane^13–15^, in proteoliposomes^16^, and when affinity-purified in detergent micelles at appropriate detergent concentrations (0.01–0.03% DDM for NhaA) (Fig. 2). The crystal structure^17^ has elucidated some of the properties of the interface between the monomers (Fig. 1a,b): at the periplasmic side of the interface, two β-hairpins of the two monomers join in an antiparallel β-sheet; at its cytoplasmic side, only a few contacts exist between TMs VII and IX of the two monomers. Lipids have been suggested to be present between these contacts^17^.

Notably, the crystal structure of NhaA does not reveal the interfacial lipids; indeed, many crystal structures of membrane proteins do not enable lipids to be identified, because of low resolution and/or delipidation caused by detergents. Therefore, many open questions remain regarding the interactions between membrane lipids and membrane proteins. Recently, a mass spectrometry platform was developed to simultaneously determine the presence of interfacial lipids and oligomeric stability^18^. In an analysis of 125 α-helical oligomeric membrane proteins, 12 membrane proteins with high oligomeric stability were shown to lack interfacial lipids. In contrast, lipids were suggested to be essential for the dimerization of two dimeric secondary transporters: NhaA^17^ and LeuT^19^, which showed the lowest oligomeric stability^18^. A separate study has shown that cardiolipin is a major component of the interfacial lipids in both NhaA and LeuT^20^. However, the details of the NhaA-cardiolipin interaction and the role of this interaction in NhaA dimerization, stability, and functionality have not yet been determined. Herein we seek to address these questions.

## Results

### Native NhaA dimers split into monomers in the presence of increasing DDM concentrations

**Figure 3:**
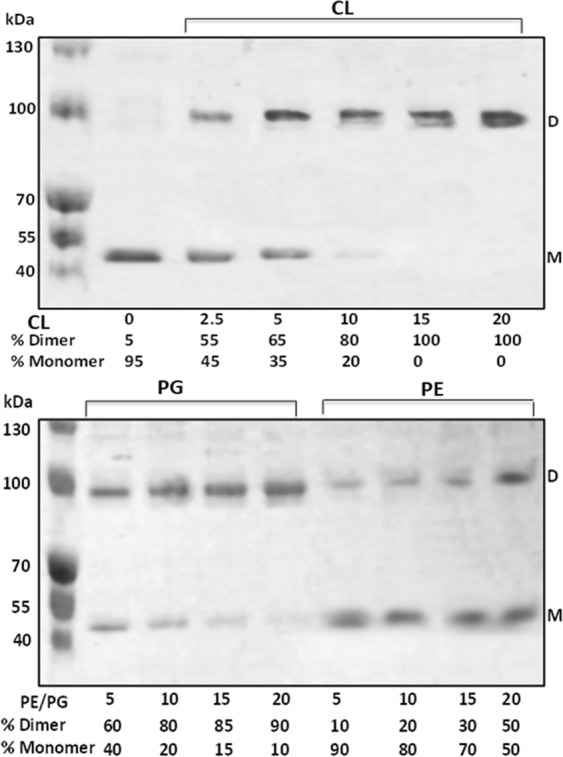
Cardiolipin efficiently reconstitutes NhaA dimers from monomers. Samples of monomeric NhaA suspension (12 µL, 1.7 µg protein, 7.6 µM NhaA) were prepared. One sample with no addition served as a control, and to the other samples different phospholipids were added at the indicated phospholipid/NhaA molar ratios: cardiolipin (CL) or L-a-Phosphatidylglycerol (PG) or L-a-phosphatidyl ethanolamine (PE). The phospholipid-containing samples were sonicated for 10 s in a bath sonicator at 23 °C and incubated for 45 min at 4 °C with slow agitation. Then, 15 µL of native gel sampling buffer was added, and the proteins were resolved on native gels. The experiment was conducted three times with identical results.

To study the cardiolipin-NhaA interaction, we first looked for conditions that delipidate NhaA. Prior studies have shown that DDM, like other detergents, can delipidate membrane proteins^21^. Moreover, intermolecular cross-linking studies have shown that DDM may affect the integrity of the NhaA interface in particular^22^ suggesting that interfacial lipids may be important for NhaA dimerization, such that exposure to DDM may delipidate the interface and split NhaA dimers into monomers. Accordingly, we prepared affinity-purified His-tagged NhaA in 0.015% DDM and pre-incubated it in the presence of increasing concentrations of DDM (Fig. 2). Then, the proteins were again affinity-purified, resolved on native gels, and stained, and the band densities were determined. After pre-incubation in up to 0.1% DDM, only dimers were observed (Fig. 2); however, after pre-incubation at 1% DDM and above, the quantity of dimers progressively decreased, whereas the quantity of monomers increased. Pre-incubation at 3% DDM yielded 85% monomers and 15% dimers, whereas pre-incubation at 5% DDM yielded 95% monomers (Fig. 3 top panel, first lane). These observations strongly suggest that, as expected, DDM delipidates the NhaA dimers, and that this delipidation extracts lipids that are needed for maintaining the dimers.

### Cardiolipin efficiently reconstitutes NhaA monomers into dimers

*E. coli* membranes are composed of ∼75% phosphatidyl-ethanolamine (PE), ∼20% phosphatidyl-glycerol (PG), and ∼5% cardiolipin (CL), where only the latter has been shown to bind in the dimer interface of NhaA^18^. We examined whether addition *in vitro* of each of these three phospholipids to samples of affinity-purified NhaA monomers, prepared by pre-incubation in 5% DDM, would reconstitute the NhaA dimers. One sample served as a control with no phospholipid addition and contained 95% monomers (Fig. 3, top panel, 0 CL). In parallel, to each remaining sample we added CL, PG or PE in increasing concentrations as indicated by molar ratio (lipid/NhaA). We incubated each sample for 45 min at 4 °C with slow agitation and subsequently resolved the proteins on native gels. Addition of CL to NhaA monomers efficiently reconstituted NhaA dimers Fig. 3. Whereas about 95% monomers were observed in the control sample with no CL, increasing the CL/NhaA molar ratio from 2.5 to 15 progressively reconstituted the monomers, ultimately yielding 100% dimers. PG was apparently less efficient: A molar ratio of 15 PG/NhaA yielded 85% dimers. However, at molar ratio twice that of CL (20 PG/NhaA) its effect on NhaA dimerization was similar to CL.

PE was much less effective; only 30% dimers were observed at a PE/NhaA molar ratio of 15, and a molar ratio of 20 yielded 50% dimers.

Taken together, these *in-vitro* results clearly show that cardiolipin acts as a key component of NhaA monomers’ dimeric assembly. They further show that PG can also reconstitute NhaA dimers from monomers *in* vitro and its efficiency equals that of CL when the PG/NhaA molar ratio is twice that of CL/NhaA.

### A mutant that does not synthesize cardiolipin produces a mixture of monomers and few unstable NhaA dimers

**Figure 4:**
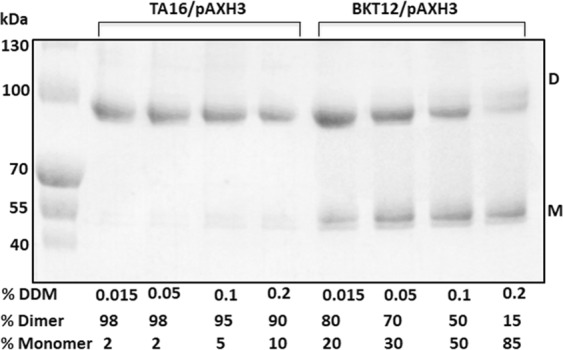
Plasmid borne *nha*A expresses a mixture of NhaA monomers and unstable dimers in BKT12, a mutant strain that does not synthesize cardiolipin. TA16/pAXH3 cells expressing WT-NhaA and a mutant that does not synthesize cardiolipin (BKT12/pI^Q^, pAXH3) bearing pAXH3 and pI^Q^ (a plasmidic combination needed for NhaA IPTG control expression) were grown on minimal medium to 0.8 OD_600_, IPTG-induced for 2 h. Then, high-pressure membranes were isolated, NiNTA affinity-purified proteins were isolated, and samples (6 µg protein/7.5 µL) were mixed with equal volumes of native gel sampling buffer, titrated to final pH 7, incubated in the indicated DDM concentrations for 10 min at 23 °C, and resolved on native gel. The experiment was conducted three times with identical results.

To investigate the NhaA-cardiolipin interaction *in vivo* in intact cells, we used an *E. coli* mutant BKT12 that does not synthesize cardiolipin^23^ and transformed it with a plasmid combination required for isopropyl β-D-1-thiogalactopyranoside (IPTG)-induced expression of NhaA (pAXH3pI^Q^). As a wild-type (WT) control we used the *E. coli* derivative TA16 transformed with the plasmid pAXH3 (TA16 carries I^Q^ on its chromosome) (see Materials and Methods). After the cells were grown and induced in minimal medium, we isolated high-pressure membrane vesicles, affinity-purified the proteins, incubated them in the presence of various DDM concentrations for 10 min, and resolved them on native gels (Fig. 4). The WT (TA16) cells primarily produced stable NhaA dimers: Pre-incubation of the proteins in up to 0.2% DDM yielded 90% dimers and 10% monomers (Fig. 4). In marked contrast, mutant BKT12 produced a mixture of about 80% dimers and 20% monomers already following pre-incubation with 0.015% DDM. The latter dimers were less stable than those produced in the WT cells: Increasing the DDM concentration above 0.015% progressively split them into monomers. For example, in BKT12-produced NhaA, pre-incubation in 0.2% DDM—which hardly affected NhaA dimers expressed from TA16—yielded a mixture comprising 85% monomers (Fig. 4). Hence, in the absence of CL, some unstable NhaA dimers are produced together with NhaA monomers.

### The mutant BKT12, which does not synthesize cardiolipin and produces a mixture of NhaA dimers and monomers, is salt-sensitive although its everted membrane vesicles exhibit antiporter activity similar to that of the wild type

**Figure 5:**
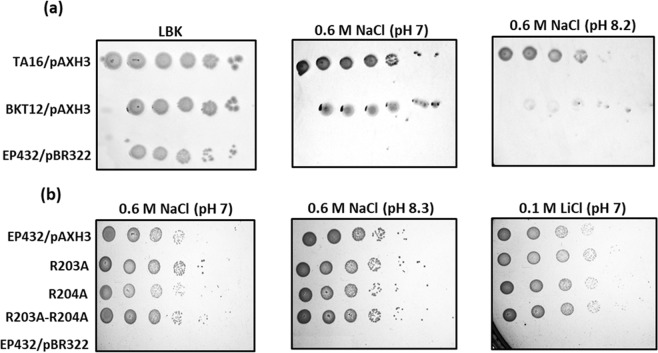
The growth phenotype conferred by WT NhaA when expressed in a host strain that does not synthesize cardiolipin and by alanine replacement mutants R203A or R204A or R203A-R204A expressed in a strain synthesizing cardiolipin. (**a**) Cells of BKT12/pAXH3, pI^Q^, a mutant host that does not synthesize cardiolipin, bearing a plasmid combination expressing NhaA, were grown on non-selective agar plates (LBK) and on the indicated selective agar media for 48 hr at 37 °C as described in the Materials and Methods section. TA16/pAXH3 expressing WT-NhaA served as a positive control, and EP432/pBR322 served as a negative control. (**b**) Cells (OD_600_ 0.5) of NhaA mutants, R203A, R204A and R203A-R204A in EP432 were tested for Na^+^/Li^+^ growth sensitivity as described in (**a**). EP432/pBR322 and EP432/pAXH3 served as negative and positive controls, respectively. (**b**) The experiments were conducted three times with identical results.

The growth phenotype of BKT12/pI^Q^/pAXH3 in the presence of high Na^+^/Li^+^ selective media was tested in comparison to a positive control (TA16/pAXH3) that expresses WT NhaA, and in comparison to a negative control (EP432/pBR322) bearing the empty vector (Fig. 5a). Under non-selective conditions (LBK), the three strains showed similar growth phenotypes. In the presence of 0.6 M NaCl at pH 7, the positive control and BKT12/pIQ/pAXH3 grew to the same extent. In the presence of 0.6 M NaCl at pH 8.2, however, whereas the positive WT control grew, the mutant could not confer salt resistance, and very few colonies were observed. As expected, the negative control did not grow in any of the selective media.

**Figure 6:**
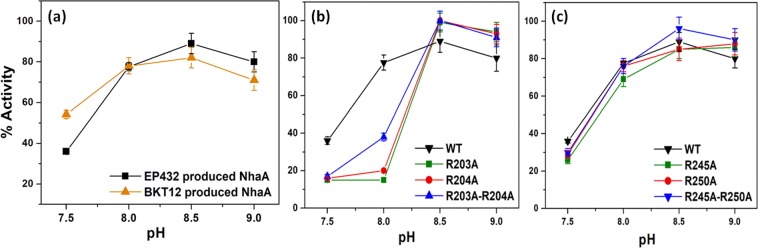
Na^+^/H^+^ antiport activity in isolated everted membrane vesicles of NhaA variants. (**a**) Plasmid borne WT *nha*A expressed in *E. coli* strains synthesizing cardiolipin (EP432) (black line) or BKT12 deprived of cardiolipin (orange line) (**b**) NhaA mutants expressed in EP432 cells, R203A, R204A and R203A-R204A (**c**) Mutants R245A, R250A and R245A-R250A. Everted membrane vesicles were prepared from cells expressing the indicated NhaA variants grown in LBK (pH 7). Na^+^/H^+^ antiport activity was determined at the indicated pH values using acridine orange fluorescence to monitor ΔpH in the presence of 10 mM NaCl (see Materials and Methods). Na^+^/H^+^ antiport activity expressed in percentage of dequenching following the addition of 10 mM NaCl is shown versus external pH. The SD is shown in bars.

The Na^+^/H^+^ antiporter activity in everted membrane vesicles of WT NhaA (pAXH3) expressed in a WT *E. coli* strain (EP432) was compared to its activity when expressed in the strain BKT12, which cannot synthesize cardiolipin (Fig. 6a). Interestingly, although the growth phenotype was different (Fig. 5a), the antiporter activity and its pH dependence were very similar when NhaA had been expressed in both *E. coli* strains. This apparent discrepancy stems for the following facts: for both tests (growth phenotype and antiporter activity in isolated membrane vesicles) the BKT12 and WT cells are grown on LBK. Yet the WT cells possess the NhaA native dimer whereas BKT12 bears a mixture of monomers and aberrant dimers (Fig. 4). As previously shown^16^ NhaA monomers are functional. This is why they show WT antiporter activity (Fig. 6). However, under selective conditions the NhaA native dimer are much better than the monomer in rendering salt resistance of growth as shown in (Fig. 5).

### The putative binding site of cardiolipin

In the NhaA dimer crystal structure^17^ an electron density was identified involving the two arginines of TM VII (at sites 203 and 204); these two arginines constitute a contact point between the two monomers of the NhaA dimer, and the electron density has been suggested to reflect the presence of lipids at that contact point (Fig. 1). To investigate whether the two arginines are indeed a lipid binding site, we mutated each arginine to alanine separately and both together and explored the mutants’ growth phenotypes on selective Na^+^/Li^+^ media (Fig. 5b). The growth phenotypes of the arginine mutants (R203A, R204A and R203A-R204A) were very similar to the WT growth phenotype on Na^+^/Li^+^ selective media. Moreover, in everted membrane vesicles, the Na^+^/H^+^ antiport activity and even the apparent *K*_m_ for Na^+^ were very similar to those of the WT at pH 8.5; the only difference observed in the mutants was an alkaline shift of about 0.5–1 unit in the pH dependence of antiport activity (Fig. 6a). These observations suggest that, *in vivo* and in the membrane, the mutants are quite stable and functional. However, *in vitro*, the mutant R203A-R204A aggregated during affinity purification, implying that the double-mutant protein is unstable. These results suggest that R203 and R204 comprise the putative cardiolipin binding site. To further support this suggestion, we performed molecular docking study (Supplementary Information and Fig. S1). The anionic characteristics of CL (due to the presence of two phosphate groups) favored the cationic two arginine moieties, i.e., Arg203 and Arg204.

**Figure 7:**
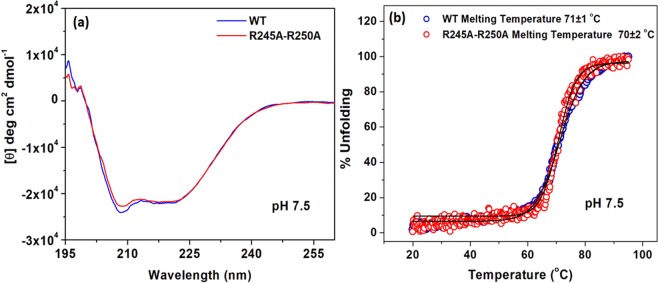
CD spectra of WT NhaA and mutant R245A-R250A. The respective proteins (4 µM) were affinity-purified and re-suspended in dialysis buffer titrated to pH 7.5 with Tris base. The CD spectra were recorded on a JASCO J-810 CD Spectro-polarimeter (JASCO, Inc., Japan), using the supplied Spectra Manager software. (**a**) Comparison of CD spectra for the WT and mutant R245A-R250A at 4 °C. (**b**) Unfolding transition midpoint (T_m_) values were determined from the corresponding inflection points. Traces are representative from three different measurements.

At the N-terminus of TM IX, in close proximity to R203 and R204, reside two additional arginines, R245 and R250 that can also bind cardiolipin (Fig. 1a)^24^; we mutated these arginines, too, to investigate whether they might contribute to the cardiolipin binding site. The three mutants we produced—R245A, R250A and R245A-R250A—were similar to the WT in terms of the growth they elicited in selective media and their antiport activity in everted membrane vesicles (Fig. 6b). Furthermore, circular dichroism (CD) analysis showed that the R245A-R250A protein was as stable as the WT (Fig. 7). These results imply that R245 and R250 are not involved in the cardiolipin binding site.

## Discussion

Recent mass spectrometry experiments have shown that the phospholipid cardiolipin is bound in the dimer interface between the monomers of NhaA^18^. Here, we explored the cardiolipin-NhaA interaction *in vitro* and *in vivo* and showed that cardiolipin is essential for NhaA dimerization and for dimer stability, as well as for NhaA functionality.

### Cardiolipin efficiently reconstitutes dimers from DDM-delipidated NhaA monomers *in vitro*, whereas PG and PE are less efficient

We first showed that, whereas affinity-purified NhaA proteins in DDM (0.015%-0.03%) micelles persist as dimers (Fig. 2), increasing the DDM concentration to 1% or above progressively splits the NhaA dimers into monomers. As detergents are known to delipidate membrane proteins^21^, these results strongly suggest that a high concentration of DDM delipidates phospholipids that are essential for maintaining NhaA dimers. We then tested the capacity of each of the three phospholipids that are present in the *E. coli* membrane (PE, PG, and CL, present at concentrations of ~75%, ~20%, and ~5%, respectively) to reconstitute dimers from NhaA monomers produced by DDM exposure. Strikingly, though CL is the least abundant phospholipid in the membrane, addition of CL *in vitro* to delipidated NhaA monomers (CL/NhaA molar ratio of 15) reconstituted 100% of the monomers into dimers. PG, a major phospholipid was essentially as efficient as CL if we consider that CL is a dimer of phosphatidic acids connected by a glycerol; At molar ratios of 20 phospholipid/NhaA, PG reconstituted 90% of the monomers into dimers as did CL at a molar ratio of 10/NhaA.

Nevertheless, the atomic structure of 2PG bound versus 1CL bound can be different and the former less stable. Indeed, this was hinted in the results summarized in Fig. 4. The dimers produced, possibly by PG, in a strain (BKT12) that does not synthesized CL, are less stable than when produced by the WT. PE, the other major phospholipid was much less efficient in the *in-vitro* reconstitution test (Fig. 3). We conclude that CL is essential for the dimerization and dimeric structure of NhaA, though PG can fulfill this function when its concentration is twice that of CL. Notably, these results also point to a novel means of exploring interactions between oligomeric membrane proteins and lipids that are proposed to be present at their interface: simply testing which phospholipids reconstitute oligomers of delipidated affinity-purified membrane proteins *in vitro*.

### Cardiolipin is required for stability of NhaA dimers

As noted in the introduction, the crystal structure of the NhaA dimer^17^ revealed the structural elements that connect the monomers. These include the β-hairpin that forms the β-sheet at the periplasmic side of the NhaA dimer and the interactions between TM VII of one monomer and TM IX of the other monomer at the cytoplasmic side (Fig. 1). Subsequent studies confirmed that each of these elements is crucial for the NhaA dimer structure: mutations in which one or both elements were deleted, Δ(β-hairpin)^16^, Δ(VI-VII), and Δ(β-hairpin, VI-VII))^16,25^ yielded monomeric NhaA. Surprisingly, the three mutations produced different growth phenotypes on routinely used salt-selective media (0.6 M NaCl at pH 7 or pH 8.3 and 0.1 M LiCl at pH 7): The Δ(β-hairpin) mutant grew similarly to the WT^16^, and its Na^+^/H^+^ antiport activity, measured in inside-out membrane vesicles, was very similar to that of the WT in terms of rate, pH profile and apparent *K*_M_ for Na^+^. These observations imply that the functional unit of NhaA is the monomer; indeed, the benefit of the dimer over the Δ(β-hairpin) monomer was revealed only under extreme stress conditions, when the WT dimers conferred salt resistance, whereas the monomers did not^16,26^. In contrast, the Δ(VI-VII) and Δ(β-hairpin, VI-VII) mutants could not grow at pH 8.3 in the presence of 0.6 M NaCl. Taken together, these observations suggest that both the β-hairpin and another factor involving TMs VI and VII are needed for NhaA dimeric structure and stability, and that the latter factor may also be necessary for functionality. According to the results presented herein (Fig. 3), this factor is likely to be cardiolipin.

Instead of producing NhaA monomers through mutagenesis of the protein, we used BKT12/pAXH3*/pIQ*, a host cell that does not synthesize cardiolipin^23^. This strain produced a mixture of NhaA monomers and dimers (Fig. 4). The appearance of the dimers in BKT12/pAXH3*/pIQ* can be accounted for by the presence of PG in the cardiolipin-less mutant; as shown herein, PG can reconstitute NhaA dimers when cardiolipin is absent (Fig. 3). It would be interesting to investigate a mutant lacking both PG and CL.

Notably, the dimers produced in the absence of cardiolipin were less stable than WT dimers (Fig. 4): Whereas affinity-purified dimers of the WT protein remained stable in DDM concentrations of up to 0.1%, the dimers produced in BKT12 were sensitive already to 0.015% DDM, and the proportion of monomers progressively increased as we increased the detergent concentration. At 0.2% DDM 85% monomers were observed (whereas the proportion of monomers for WT NhaA at this DDM concentration was only 10%) (Fig. 4). We conclude that CL is an optimal phospholipid for the stability of NhaA dimers.

Surprisingly, whether expressed in EP432 or BKT12 *E coli strains* the antiporter activity of NhaA in everted membrane vesicles was very similar (Fig. 6a). For preparing everted membrane vesicles the cells were grown in non-selective medium (LBK) and under this condition the activity of NhaA is dispensible so both BKT12 and EP432 expressing NhaA (with or without CL) grew alike. On the other hand, under stress conditions of growth the instability of NhaA deprived of CL compromised growth.

Notably, the growth phenotype of BKT12/pAXH3*/pI*^*Q*^ was very similar to the growth pattern of the monomeric mutants Δ(VI-VII) and Δ(β-hairpin and VI-VII) (compare Figs. 5a to 2 in^25^). Specifically, in the presence of 0.6 M NaCl, BKT12/pAXH3*/pI*^*Q*^, like Δ(VI-VII) and Δ(β-hairpin and VI-VII), hardly grew at pH 7 and did not grow at pH 8.2 (Fig. 5a). The common denominator of these three salt-sensitive strains is a lack of CL: CL is not produced in BKT12/pAXH3*/pI*^*Q*^, and the CL binding site is likely to be abrogated in the mutants deleted of TMs VI-VII (see below).

Very few studies of the interaction between transporters activity and lipids have been done^27,28^, recent extensive study on lipids - melibiose permease (MelB) interaction, using a BKT strain, showed that CL is not needed for MelB folding, stability, and activity^27^. Unlike the dimeric NhaA, MelB is a monomer. Therefore, these results may support the idea that CL is involved for a dimer formation.

### The putative binding site of cardiolipin

Given that cardiolipin is present in the NhaA dimer interface, the NhaA dimer crystal structure may provide clues regarding the location of the cardiolipin binding site^17^ (Fig. 1a,b). Specifically, the dimer structure contains a large pear-shaped space that separates the two monomers; this structure has an apex at L255 of TM IX and is suggested to be full of lipids^17^. L255 and residues above it toward the cytoplasm are in close proximity to their twins on the other monomer, and single-Cys replacements of these residues have been shown to form intermolecular cross-linking across the dimer interface^22^ (Fig. 1a). Interestingly, the twin L255C is likely to be involved in a rigid intermolecular interaction, as it cross-linked only with the short cross-linker 1,2-ethanediyl bismethanethiosulfonate (MTS-2-MTS) (spanning 5 Å) or formed an S-S bond^22^. Toward the β-hairpin-loop (Fig. 1a,b) at the periplasm, W258 on TM IX of one monomer makes a bridge to TM VII of the other monomer, and V254 on TM IX of one monomer interacts with R204 of TM VII of the other monomer. Thus, the dimer interface above and below the pear-shaped interspace is bordered (or sealed) by interactions between TMs (VII and IX of each monomer). Where, then, does the CL interact in the dimer interface?

CL with its two phosphates is a potential anion^29^. It was demonstrated recently that CL head-group is fully ionized as a dianion and CL behave as strong dibasic acid within the physiological pH^30^. Therefore, it is likely that positively charged residues contribute to its binding site. As noted above, in the NhaA dimer crystal structure^17^, an electron density was identified in the vicinity of the four arginines at the TM VII–TM VII contact point of the two monomers (two arginines in each monomer; R203 and R204; Fig. 1)^17^, and this density was later suggested to indicate the presence of CL^31^. In *in-vivo* experiments, alanine replacements of these arginines, R203A, R204A and R203A-R204A, grew on selective media similarly to the WT (Fig. 5b), and in membrane vesicles the only difference observed with respect to the WT was an alkaline shift in Na^+^/H^+^ antiport activity (Fig. 6b). In contrast, *in vitro*, the protein R203A-R204A aggregated during affinity purification. In other words, whereas the mutant lacking the arginines R203A and R204A is functional *in vivo*, the protein is unstable *in vitro*. This difference can be accounted for by the fact that PG, which can constitute NhaA dimers (albeit less efficiently than cardiolipin see above) was present in our *in-vivo* experiments but not in our *in-vitro* experiments. The involvement of Arg203 and Arg204 in the putative CL binding site was supported by using molecular docking study (Supplementary Information and Fig. S1). Taken together, these results imply that the TM VII-arginines contribute to the CL binding site, which is needed for dimerization and stability of NhaA.

We also investigated whether residues R245 and R250, which are in close proximity to the putative cardiolipin binding site (Fig. 1a)^24,31^, might also contribute to cardiolopin binding. We determined that they do not: Mutants R245A, R250A, and R245A-R250A grew on selective media and exhibited antiport activity similar that of the WT (Fig. 6c). Furthermore, the affinity-purified protein R245A-R250A was stable and showed CD spectra similar to those of the WT (Fig. 7).

### The role of cardiolipin in NhaA functionality

Our *in-vivo* experiments, in which CL-less NhaA did not confer salt resistance, indicated that CL is important for NhaA functionality. Yet, our previous studies of the monomeric mutant NhaA-Δβ revealed that the NhaA monomer is the functional unit of NhaA^16^. What, then, is the functional role of cardiolipin in NhaA?

First, it is important to note that several studies have indicated that functional interactions take place between NhaA monomers^14,32^. Particularly compelling is the observation that mutations of residues in or near the regions proposed herein to constitute the CL binding site (Fig. 1) affect NhaA Na^+^/H^+^ antiport activity^14^. Specifically, the following effects were observed: (a) Inter-molecular cross-linking of a single NhaA-V254C mutant by a rigid cross linker dramatically changed the pH profile of cysteine-less NhaA/V254C, whereas a long and flexible cross-linker had no effect^14^. (b) Chemical modification of L255C increased the apparent *K*_m_ for Na^+^ 6-fold and changed the pH profile by 1 unit to the alkaline side^22^. (c) Cys replacements of the potentially positively-charged residues in this segment of NhaA, K242C, R245C, K249C, R250C, and H256C caused an increase (4–10-fold) in the apparent *K*_m_ for Na^+^ ^22^. Interestingly, K249 is the only trypsin-cleavable site of NhaA^33^, and the pH profile of the trypsin cleavage reflects the pH dependence of the antiport activity^34^.

Recent experiments that we have carried out may shed additional light on the functional role of CL in NhaA. We adapted hydrogen/deuterium-exchange mass spectrometry (HDX-MS) to identify global conformational changes in NhaA upon Li^+^ binding at physiological pH^35^. Our analysis revealed a pronounced Li^+^-induced deuterium uptake-change in TMs IX at the CL binding site. It is possible that, for this conformational change, CL may be required. Hence, the observation that the NhaA monomer is a functional unit does not preclude the possibility that CL is needed for functionality, and moreover, CL interaction in the dimer may contribute to fine tuning of antiport activity. This proposition is consistent with the observation that genes for CL synthesis are also upregulated in *E. coli* under salt stress^36^, implying that CL might have a regulatory role.

Thus, the presence of CL in the dimeric interface between the NhaA monomers is not related to the structural fold, nor is it necessary for Na^+^/H^+^ antiport activity; rather, its primary role seems to be conferring stability to the dimer and regulating conformational changes. Indeed, as noted above, CL has also been identified in the dimer interface of LeuT^18,20^, a transporter whose structural fold is different from that of NhaA^8^, yet that shows similarly low dimeric stability. In contrast, NapA, the Na^+^/H^+^ antiporter of *Thermus thermophilus*, which shares the NhaA fold, does not specifically bind phospholipids, and shows high dimeric stability^20^. The stability of NapA stems from its structure, an additional N-terminal helix that is absent from NhaA and that strengthens the monomer–monomer interface. Taken together, these observations suggest that different membrane proteins use different ‘tools’ for preserving their oligomeric states and regulating conformational changes: Some rely on structural elements that ensure tight contact between subunits, whereas others recruit lipids for this purpose. These insights raise the intriguing question of whether the human NhaA homologues that are involved in disease also require specific phospholipids. If they do, their phospholipid binding sites may be effective drug targets.

## Materials and Methods

### Plasmids, bacterial strains, and culture conditions

pAXH3^16^ is a plasmid expressing His-tagged NhaA. The plasmid pI^Q^ is a pACYC184 derivative encoding the lac repressor I^Q^ needed for controlled induction of NhaA by IPTG. EP432 is an *E. coli* K-12 derivative, which is *mel*BLid, Δ*nha*A1::kan, Δ*nha*B1::cat, Δ*lac*ZY, *thr*1^37^. TA16 is an *E. coli* K-12 derivative (*nha*A^+^, *nha*B^+^, *lac*I^Q^) and is otherwise isogenic to EP432^16,34^. BKT12 is an *E. coli* strain that was obtained from Yale *E. coli* Genetic Stock Center #13593 (6F- *λ*^*−*^, *ΔclsB861*, *ΔclsC788::kan*, *ΔclsA856*, *IN(rrnD-rrnE)1*, *rph-1*). BKT12/PI^Q^/pAXH3 is BKT12 transformed with pI^Q^ and pAXH3.

Cells were grown either in Luria broth (LB) or in modified LB (LBK) in which NaCl was replaced with KCl. The medium was buffered with 60 mM 1,3-Bis[tris(hydroxymethyl)methylamino]propane (BTP). For plates, 1.5% agar was used.

To test cell resistance to Li^+^ and Na^+^, EP432 or TA16 cells transformed with the respective plasmids were grown on LBK to A_600_ of 0.5. Samples (2 µl) of serial 10-fold dilutions of the cultures were spotted onto agar plates containing the selective media: modified LB in which NaCl was replaced with the indicated concentrations of NaCl or LiCl at the various pH levels and incubated for 2 days at 37 °C.

### Site-directed mutagenesis

Site-directed mutagenesis was carried out according to a polymerase chain reaction-based protocol^38^ with pAXH3 as a template. All plasmids carrying mutations are designated by the name of the plasmid followed by the mutation.

### Overexpression and affinity purification of NhaA proteins

Overexpression of NhaA^33^, isolation of high-pressure membranes^39^, and affinity purification (Ni^2+^-nitrilotriacetic acid-agarose, Qiagen)^40^ were performed as described previously, but the protein was eluted in a buffer (pH 7.5) containing 300 mM imidazole, 25 mM citric acid, 100 mM choline chloride, 5 mM MgCl_2_, and 0.015% n-dodecyl β-D-maltopyranoside (DDM). Sucrose (10%) was added to the eluted protein solution, and the solution was dialyzed overnight at 4 °C in acidic dialysis buffer containing 10% sucrose, 100 mM choline chloride, 25 mM citric acid, 5 mM MgCl_2_, 0.015% DDM (pH 4.0), and was frozen at −80 °C.

### Detection and quantification of NhaA

Total quantities of membrane and affinity-purified proteins were determined using the Bradford assay^41^. In certain cases, quantitation of the affinity-purified NhaA was carried out by resolving the sample and a sample of known NhaA concentration on SDS-PAGE, staining the gels with Coomassie Blue and quantifying the band densities using Image Gauge (Fuji) software^42^.

### *In-vitro* reconstitution of NhaA dimers from monomers by cardiolipin

To obtain NhaA monomers, affinity-purified NhaA in the dialysis buffer was diluted 3 folds into potassium phosphate (KPi) buffer containing 100 mM KPi (pH 7.5), 5 mM MgCl_2_ and 5% DDM. The mixture was incubated at 4 °C for 90 min in Eppendorf thermomixer (Comfort) with slow agitation (300 rpm). Then, the protein was loaded on NiNTA, thoroughly washed in KPi buffer containing 0.015% DDM (X3 times 1. 5 mL) to get rid of the high detergent concentration, and eluted in elution buffer. Then, samples of the monomeric eluted suspension (12 µL, 1.7 µg protein, 7.6 µM NhaA) were prepared. One sample with no addition served as a control, and to the other samples different phospholipids were added at different phospholipid/NhaA molar ratios: cardiolipin (Avanti, 841199), PG, (Avanti, 841188) or PE (Avanti, 840027). The phospholipid-containing samples were sonicated for 10 s in a bath sonicator (Laboratory Supplies Co. USA Model G 112 SPIT, 600 V/Ts, 80KC, 5 Amp) at 23 °C and incubated for 45 min at 4 °C with slow agitation. Then, 15 µL of native gel sampling buffer was added, and the proteins were resolved on native gels to analyze the percentages of monomers and dimers.

### Blue native-PAGE (BN-PAGE)

BN-PAGE was carried out as previously described^43^ with small modifications for NhaA^16,26^. The main gel and the overlay were made of 10% and 4% polyacrylamide, respectively, 25 mM imidazole/HCl pH 7, 0.5 M 6-amino caproic acid and 0.015% DDM. The sample buffer contained the protein and 50 mM NaCl, 1 mM EDTA, 50 mM imidazole/HCl, pH 7, 0.015% DDM, and 5% glycerol. The cathode buffer contained 50 mM Tricin, 7.5 mM imidazole/HCl (pH 7), 0.015% DDM and Coomassie Blue 0.02% (G250; Merck). The anode buffer contained 25 mM imidazole /HCl (pH 7) and 0.015% DDM. The electrophoresis was conducted at 15 mA for 1 h. The gel was stained by Coomassie Blue and dried, and the band densities were determined as above.

### Isolation of membrane vesicles and assay of Na^+^/H^+^ antiport activity

EP432 cells transformed with the respective plasmids were grown in LBK medium, and everted membrane vesicles were prepared and used to determine the Na^+^/H^+^ antiport activity as described previously^44,45^. The assay of antiport activity was based upon the measurement of Na^+^-induced changes in ΔpH as measured by acridine orange, a fluorescent probe of ΔpH. The fluorescence assay was performed with 2.5 ml of reaction mixture containing 50–100 µg of membrane protein, 0.5 µM acridine orange, 150 mM cholineCl, 50 mM BTP, and 5 mM MgCl_2_, and the pH was titrated with HCl as indicated. After energization with D-lactate (2 mM, pH7 titrated by KOH), quenching of the fluorescence was allowed to achieve a steady state, and then Na^+^ (10 mM) was added. A reversal of the fluorescence level (dequenching) indicates that protons are exiting the vesicles in antiport with Na^+^. As shown previously, the end level of dequenching is a good estimate of antiport activity^46^, and the concentration of the ion that gives half-maximal dequenching is a good estimate of the apparent *K*_m_ for Na^+^ (or Li^+^) of the antiporter^46,47^. The concentration-range of the cations tested was 0.01–100 mM at the indicated pH values, and the apparent *K*_m_ values were calculated by linear regression of the Lineweaver-Burk plot.

## Supplementary information

Figure S1
