# Supplementary material for: Cardiolipin is an Optimal Phospholipid for the Assembly, Stability, and Proper Functionality of the Dimeric Form of NhaA Na+/H+ Antiporter
Source: Sci Rep. 2019 Nov 27;9:17662. doi: 10.1038/s41598-019-54198-8 (PMC6881326; doi:10.1038/s41598-019-54198-8)
Supplement: Supplementary file 1 — Figure S1 [file 41598_2019_54198_MOESM1_ESM.docx]

**Supplementary Information for**

**Cardiolipin is an Optimal Phospholipid for the Assembly, Stability, and Proper Functionality of the Dimeric Form of NhaA Na^+^/H^+^ Antiporter**

Rimon Abraham^1†^, Ramakanta Mondal^1†^, Assaf Friedler^2^ and Etana Padan^1^*

^1^Department of Biological Chemistry, Alexander Silberman Institute of Life Sciences, and ^2^Institute of Chemistry, the Hebrew University of Jerusalem, Edmond J. Safra Campus, Givat Ram, Jerusalem-91904, Israel

*Corresponding author: Etana Padan email: [etana@vms.huji.ac.il](mailto:etana@vms.huji.ac.il)

^†^These authors contributed equally.

**Experimental**

***Molecular Docking Study.***

The docking study was performed using AutoDock 4.2 suite^S1,S2^ of programs which utilizes the Lamarckian Genetic Algorithm (LGA). The three-dimensional structure of the native NhaA was taken from the Protein Data Bank, PDB.ID: 4ATV and the structure of cardiolipin was prepared and optimized using Gaussian 03W software package^S3^. The grid maps were calculated using AutoGrid and the grid size along X-, Y-, and Z-axis was 126, 126 and 126, respectively with 0.375 Å grid-spacing. The docking parameters used were as follows: GA population size = 150; maximum number of energy evaluations = 2500000; GA crossover mode = two points. The minimum binding energy conformer was searched out of 10 different conformations for each docking simulation. The visualization of the docked conformations was performed with PyMOL software package^S4^.

*The probable binding location of cardiolipin by molecular docking study.*

The probable binding site of CL in NhaA has been characterized from an AutoDock-based docking study. The minimum energy docked conformation which corresponds to the best energy solutions in the most populated cluster, reveals the dimer interface of the protein with the favorable binding site for cardiolipin (CL). The anionic characteristics of CL (due to the presence of two phosphate group) favored the cationic two arginine moieties, i.e., R203 and R204. The lipid binding free energy was calculated by using AutoDock which is -1.5 kcal/mol.


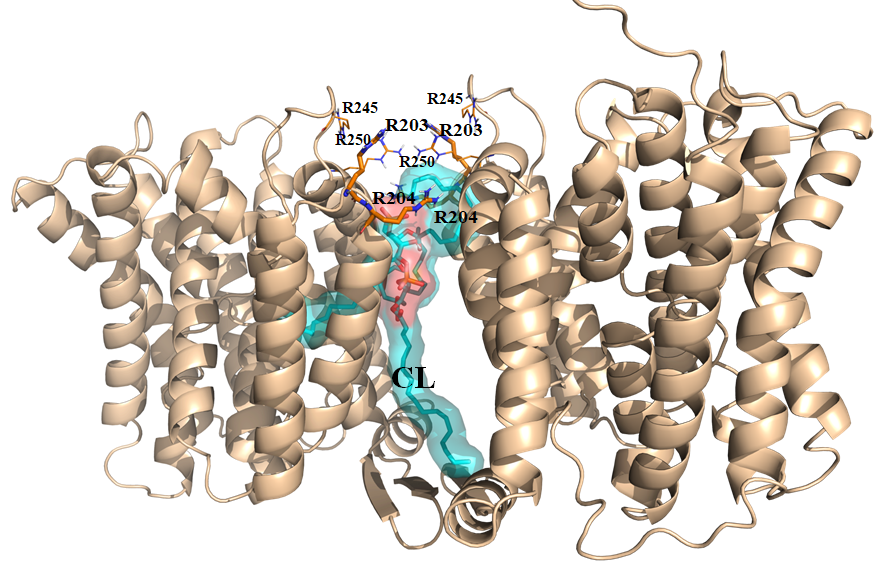


Figure S1. **The putative cardiolipin binding site in NhaA from minimum energy docked conformation by blind docking study**. The three dimensional structure of the NhaA dimer **(PDB ID: 4ATV)** is shown in cartoon representation (brown) and Arginine residues in the dimer interface are shown in stick representation (orange). The minimum energy docked pose of cardiolipin (CL) from blind docking is shown in surface model (sky blue) and phosphate groups in CL are in red. The visualization of the docked conformations was performed on PyMOL software.

# References

(S1) Morris, G. M., Goodsell, D. S., Halliday, R. S., Huey, R., Hart, W. E., Belew, R. K., Olson, A. J. Automated docking using a Lamarckian Genetic Algorithm and an empirical binding free energy function. *J. Comput. Chem.* **19**, 1639-62 (1998).

(S2) Morris, G. M., Huey, R., Lindstrom, W., Sanner, M. F., Belew, R. K., Goodsell, D.S., Olson, A.J. AutoDock4 and AutoDockTools4: automated docking with selective receptor flexibility. *J. Comput. Chem.* **30**, 2785-91(2009).

(S3) Frisch, M. J. et al. Gaussian 03, Revision B.03, Gaussian, Inc., Pitturgh, PA, 2003.

(S4) De Lano, W. L. *The PyMOL molecular graphics system*, De Lano Scientific, San Carlos, CA, USA, 2002.
